# Late Periprosthetic Joint Infection After a Total Hip Arthroplasty Caused by Peptoniphilus Species: A Case Report and Literature Review

**DOI:** 10.7759/cureus.83211

**Published:** 2025-04-29

**Authors:** Daisuke Inoue, Tamon Kabata, Yu Yanagi, Musashi Ima, Satoru Demura

**Affiliations:** 1 Department of Orthopedic Surgery, Graduate School of Medical Science, Kanazawa University, Kanazawa, JPN; 2 Department of Orthopedic Surgery, Graduate School of Medical Sciences, Kanazawa University, Kanazawa, JPN

**Keywords:** anaerobe, late periprosthetic joint infection, peptoniphilus species, total hip arthroplasty, two-stage revision

## Abstract

In this report, we present the clinical course of a 65-year-old woman who underwent a two-stage revision total hip arthroplasty (THA) for a late periprosthetic joint infection (PJI) caused by *Peptoniphilus* species (*Peptoniphilus* sp.). Furthermore, we conducted a literature review to contribute to the growing body of evidence regarding PJI caused by *Peptoniphilus* sp. Recognizing *Peptoniphilus* sp. as a potential causative pathogen in late PJI is important. Treatment should involve a two-stage revision surgery, with clindamycin (CLDM) and cephalosporins recommended for postoperative antibiotic therapy.

## Introduction

Total hip arthroplasty (THA) is an effective orthopedic surgical procedure for patients with symptomatic, advanced hip osteoarthritis. However, with the increasing number of primary THA procedures due to longer life expectancy and improved implant quality, perioperative complications following primary THA have also increased [1]. Among these complications, periprosthetic joint infections (PJIs) are particularly challenging, as they often require multiple surgical interventions to eradicate the infection, resulting in poor clinical outcomes [2]. The incidence of acute PJIs following primary THA is approximately 0.5%, while the rate of late PJIs is lower than that of acute surgical site infection (SSI) or PJI [3]. Anaerobic microorganisms account for 3%-6% of all PJIs, with most cases caused by Gram-positive cocci or Gram-negative bacilli, such as *Staphylococcus aureus* and *Escherichia coli* [4]. To the best of our knowledge, *Peptoniphilus *species (*Peptoniphilus *sp.) are extremely rare as the organism profile of PJI, meanwhile, they sometimes act as part of polymicrobial infections and are commonly found in chronic wounds and diabetic ulcers. [5].

Herein, we report the clinical course of a 65-year-old woman who underwent a two-stage revision THA for late PJI caused by *Peptoniphilus* sp. Additionally, we conducted a literature review to contribute to the growing body of evidence regarding late PJI caused by Peptoniphilus sp., as, to the best of our knowledge, this condition has been reported in only two previous studies [6,7].

## Case presentation

The patient was diagnosed with athetoid cerebral palsy. At the age of 47 years, she occasionally experienced bilateral coxalgia, although the pain did not limit her activities of daily living. By 62 years of age, the bilateral coxalgia had worsened, and the patient was referred to our hospital. Hip radiographs revealed end-stage osteoarthritis in the right hip joint and advanced osteoarthritis in the left hip joint, both due to acetabular dysplasia. A primary cementless THA of the right hip was performed via the anterolateral approach using a computed tomography (CT)-based navigation system. One month later, the left hip underwent primary cementless THA. For the acetabular component, a Trident PSL acetabular cup (Stryker, Mahwah, NJ, USA) was implanted, while an Accolade II (Stryker, Mahwah, NJ, USA) was inserted into the femoral canal. At six months postoperatively, the patient was pain-free in both hips and able to walk steadily without using a T-cane.

**Figure 1:**
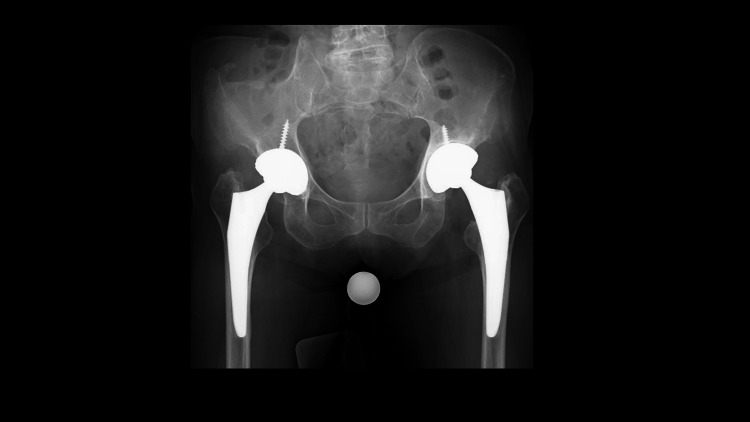
Preoperative hip radiograph A radiolucent line appears in the left acetabular implant and the shoulder of the femoral implant.

At one year postoperatively, a hip radiograph revealed a radiolucent line (RLL) around the proximal shoulder of the left femoral stem, despite the absence of bilateral hip pain. However, the patient developed left-sided hip pain upon weight-bearing as the RLL around the proximal shoulder of the left femoral stem and acetabular cup slightly expanded (Figure 1).

**Figure 2:**
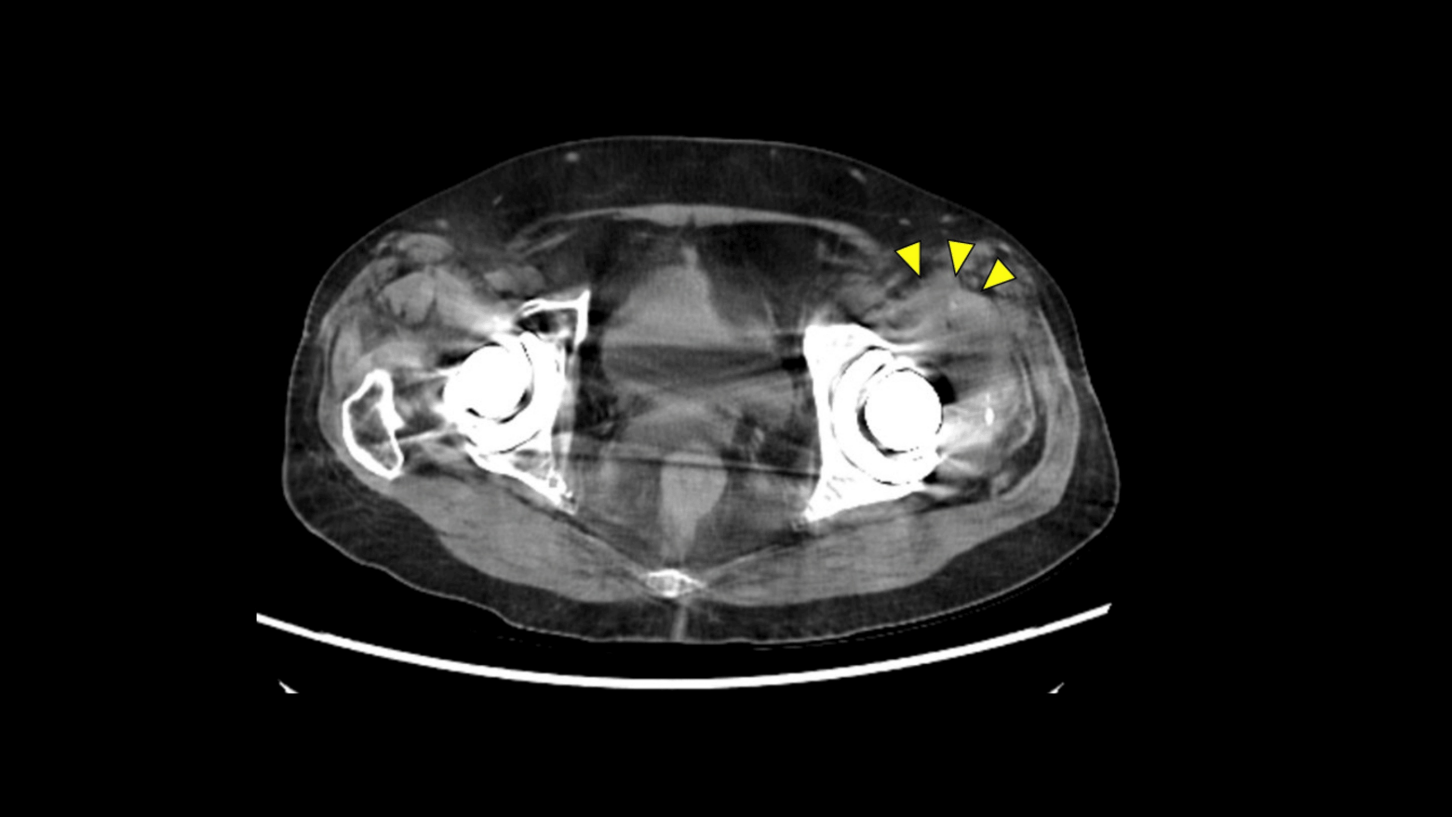
Preoperative computed tomography (CT) scan of the hip Fluid collection is observed in the left hip joint.

A hip CT revealed a fluid collection in the left hip joint. The fluid was aspirated and tested (Figure 2).

No organisms were isolated, although the leukocyte elastase test result was 3+. Routine blood test results showed a slight elevation in the values of inflammatory markers, including an erythrocyte sedimentation rate (ESR) of 54 mm/h (normal: <20 mm/h) and a C-reactive protein (CRP) level of 0.4 mg/dL (normal: <0.3 mg/dL) (Table 1).

**Table 1:** Preoperative laboratory blood results WBC: white blood cell; Hb: hemoglobin; Plt: platelet; CRP: C-reactive protein; ESR: erythrocyte sedimentation rate

Lab test	Results
WBC (/μl) (normal: 3300-8600)	6450
Neutrophil (％) (normal: 40.7-77.0)	66.4
Hb (g/dl) (normal: 11.6-14.8)	11.6
Plt (×10³) (normal: 158-348)	373
CRP (mg/dl) (normal: <0.3)	0.4
ESR (1 hr) (normal: < 20)	54

According to the 2018 International Consensus Meeting criteria, this case was inconclusive for late PJI but was suspicious of low-grade late PJI [8]. Therefore, the diagnosis was confirmed intraoperatively using an alpha-defensin test and frozen section analysis.

Operative procedure and postoperative clinical course

**Figure 3:**
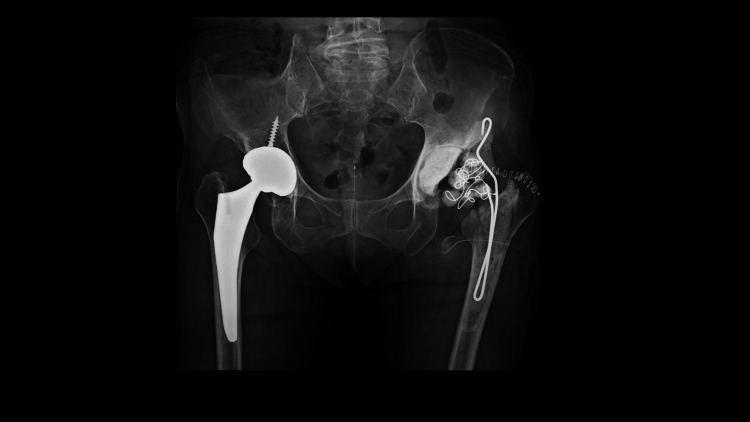
Hip radiograph from the first surgery All implants in the left hip joint were removed, and an antibiotic-loaded cement spacer was inserted into the acetabular and femoral canal.

The posterolateral approach was used with the patient in the lateral decubitus position. Fluid collection was aspirated, and the alpha-defensin test result was positive. Inflammatory synovial tissue from the hip joint and proximal femur around the femoral stem was collected to identify the causative organism. Frozen sections revealed multiple multinucleated neutrophilic infiltrates (10>400 high-power fields (HPF)). After intraoperative tissue samples and joint fluid were obtained, the acetabular cup was removed using an explant device (Zimmer, Inc., Warsaw, IN, USA), and the femoral implant was extracted using the Steinman pin technique [9]. Inflammatory and necrotic tissue was resected. Subsequently, the hip joint and femoral canal were irrigated using diluted 0.2% povidone-iodine. Finally, an antibiotic-loaded bone cement spacer containing vancomycin (6 g) and amikacin (1.2 g) was inserted into the acetabular side and femoral canal (Figure 3).

Postoperatively, *Peptoniphilus *sp. were isolated from intraoperative tissue samples and joint fluid using enrichment culture microbiological techniques [10]. Based on microbiological sensitivity results, a two-week course of antibiotic-specific therapy with intravenous clindamycin (CLDM) and cefazolin (CEZ) was initiated immediately, followed by a switch to oral CLDM and cefalexin (CEX) for four weeks. At six weeks postoperatively, laboratory results showed minimal inflammatory changes, with an ESR of 25 mm/h and a CRP level of 0.1 mg/dL. Oral antibiotic suppression therapy was continued until final reimplantation at eight weeks postoperatively.

**Figure 4:**
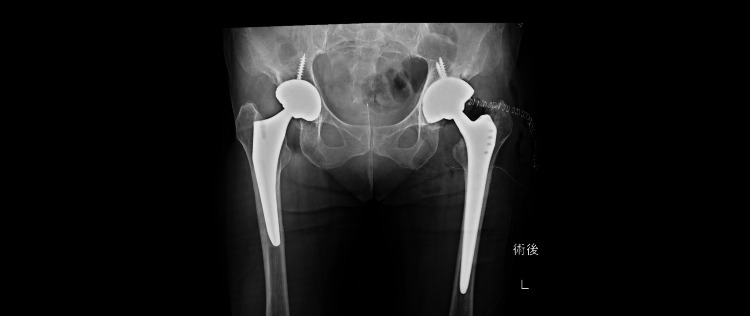
Hip radiograph from the two-stage revision surgery

In the final operation, a posterolateral approach was used with the patient in the lateral decubitus position. Intraoperatively, the soft tissues appeared in good condition, and bone stock was adequate. Tissue samples from the hip joint and femoral canal were collected for cultures and histological analysis. Frozen sections showed no evidence of multiple multinucleated neutrophilic infiltration (5≦400 HPF). Based on these findings, final reimplantation was performed. The acetabular cup (G7 Osseo-Ti multihole 58 mm, Zimmer, Inc.) was press-fitted into the acetabulum using CT-based hip navigation, followed by insertion of a Zweymüller-type long femoral stem (Alloclassic SLL, Zimmer, Inc.) into the femoral canal (Figure 4).

**Figure 5:**
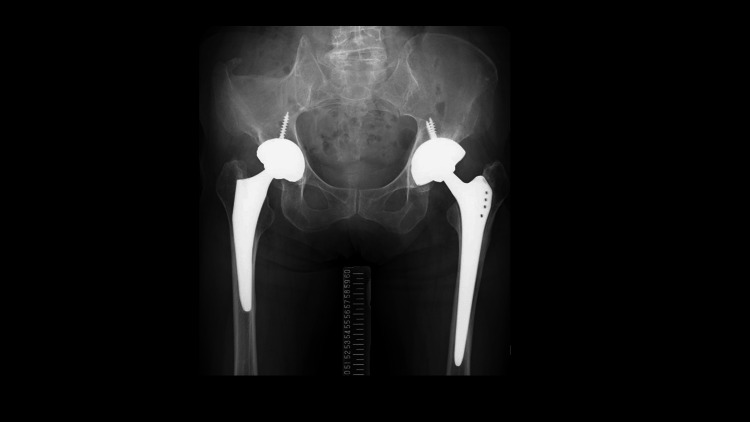
Hip radiograph obtained during the last follow-up (one year after the two-stage revision surgery)

Postoperatively, the patient was allowed full weight-bearing ambulation and received intravenous CLDM and CEZ for two weeks, followed by a switch to oral CLDM and CEX. Chronic suppression therapy was continued for six months postoperatively. At the final follow-up, one year after the final implantation, laboratory results showed no signs of inflammation, with an ESR of 12 mm/h and a CRP level of 0.1 mg/dL. Radiographs showed no RLL around the acetabular and femoral implant (Figure 5).

Finally, the patient was able to walk independently without pain or other relevant symptoms in the affected hip.

## Discussion

Late PJI after THA caused by *Peptopniphilus* sp. is considered rare, as this organism is a causative agent in polymicrobial infections associated with other infectious diseases [11]. In particular, a previous study reported that *Peptopniphilus *sp. is the most prevalent organism in the colonization of chronic wounds. In fact, only two previous studies have reported cases of late PJI after primary THA caused by *Peptopniphilus *sp. (Table 2).

**Table 2:** Reported cases of late PJI after primary THA caused by Peptopniphilus sp PJI: periprosthetic joint infection; THA: total hip arthroplasty; IV: intravenous; PO: per os; OFLX: ofloxacin; DAIR: debridement, antibiotics, and implant retention; VCM: vancomycin; CTM: cefotiam; CLDM: clindamycin; RFP: rifampicin; DAP: daptomycin; CTRX: ceftriaxone; CEX: cefalexin

Name of the study	Years	Treatment	Antibiotics	Others
Enault C et al. [6]	2020	One stage (Failed)	After one stage revision IV: OFLX After DAIR IV: VCM+CTM Po: CLDM+RFP	One-stage revision failed. Additional operation (DAIR) was done.
Sarantis M et al. [7]	2022	Two-stage	IV: DAP+CTRX; Po: CLDM	Cement spacer: tobramycin + vancomycin
Current study	2025	Two-stage	IV: CLDM+CEZ; Po: CLDM+CEX	Cement spacer: amikacin + vancomycin

Enault et al. reported a case of late PJI in a 66-year-old female patient, which included extensive osteolysis. They initially attempted a one-stage revision for late PJI; however, additional treatment, including debridement, antibiotics, irrigation, and retention, was required due to the failure to eradicate the infection [6]. Meanwhile, Sarantis et al. reported a case of late PJI in a 68-year-old female patient following primary THA. They treated the infection with a two-stage revision, which successfully eradicated the late PJI [7]. Including our report, revision surgery for this low-grade infection caused by *Peptopniphilus *sp. is considered safe using a two-stage approach.

Culture isolation is crucial for the treatment of PJI, as appropriate antimicrobial therapy cannot be administered without identifying the causative organism. Importantly, previous reports and the present case report indicate that the identification of this organism in late PJI after THA has been achieved using enrichment culture techniques, implant sonication, and the 16S rRNA gene sequencing method, rather than standard culture methods. Therefore, it is necessary to recognize in advance that this organism can be a causative agent of late PJI, including implant loosening, and to perform alternative isolation methods other than standard culture methods. Regarding antibiotic therapy, the use of CLDM may lead to favorable clinical outcomes based on previous studies and this case report, although penicillin and cephalosporin are included in the antibacterial spectrum covering *Peptopniphilus *sp. In cases of late PJI, where biofilm formation occurs around bone, soft tissue, or implants, CLDM may be a reasonable choice, as previous studies have demonstrated its effective intracellular translocation [12]. Therefore, regardless of intravenous or oral administration, CLDM may be a valuable option based on the results of microbiological sensitivity tests.

One of the limitations could be that this is a case report and a literature review. Hence, these findings cannot be generalized because of the rare condition. As a scope for future research, a longer follow-up of the treatment for late PJI is required to generalize the way to use antibiotics in terms of the recurrence of PJI. Case series with longer follow-up will be needed in order to validate and generalize these findings.

## Conclusions

We report the clinical course of a patient who underwent a two-stage revision THA for late PJI caused by *Peptoniphilus *sp., along with a review of the literature. Importantly, previous reports and the present case indicate that identification of this organism in late PJI after THA can be achieved using enrichment culture techniques, implant sonication, and the 16S rRNA gene sequencing method, rather than standard culture methods. Therefore, recognizing Peptoniphilus sp. as a potential causative organism in late PJI is crucial. According to previous papers and our findings, a two-stage revision approach is considered safe and effective for this low-grade infection caused by *Peptopniphilus *sp. We recommend that CLDM be used with cephalosporins for postoperative antibiotic therapy.
